# A novel echocardiographic method closely agrees with cardiac magnetic resonance in the assessment of left ventricular function in infarcted mice

**DOI:** 10.1038/s41598-019-40393-0

**Published:** 2019-03-05

**Authors:** Ilaria Russo, Edoardo Micotti, Francesca Fumagalli, Michela Magnoli, Giuseppe Ristagno, Roberto Latini, Lidia Staszewsky

**Affiliations:** 1 Department of Cardiovascular Research, Mario Negri Institute for Pharmacological Research IRCCS, Milan, Italy; 2 Department of Neuroscience, Mario Negri Institute for Pharmacological Research IRCCS, Milan, Italy

## Abstract

Cardiac Magnetic Resonance (CMR) is the gold standard for left ventricular (LV) function assessment in small rodents and, though echocardiography (ECHO) has been proposed as an alternative method, LV volumes may be underestimated when marked eccentric remodeling is present. In the present study we described a novel echocardiographic method and we tested the agreement with CMR for LV volumes and ejection fraction calculation in mice with experimental myocardial infarction. Sham-operated and infarcted mice, subjected to Coronary Artery Ligation, underwent ECHO and CMR. Volumes and ejection fraction were calculated by ECHO using a standard Simpson’s modified method (ECHO pLAX) or a method from sequential parasternal short axis (ECHO pSAX) acquired mechanically by translating the probe every 1 mm along the left ventricle. The mean differences ±1.96 standard deviation near to zero suggested close agreement between ECHO pSAX and CMR; contrarily ECHO pLAX agreement with CMR was lower. In addition, ECHO was three times shorter and cheaper (Relative cost difference: pLAX: −66% and pSAX −57%) than CMR. In conclusion, ECHO pSAX is a new, fast, cheap and accurate method for LV function assessment in mice.

## Introduction

Wild-type and genetically manipulated mice are widely used in experimental models of myocardial infarction to examine the characteristics of cardiac phenotype, the role of a specific protein, or to test *in vivo* the efficacy of new therapeutic approaches^1^. The functional response of the murine heart to any intervention can now be studied *in vivo* using several non-invasive imaging techniques^2^. To date, the two most widely used imaging methods are echocardiography (ECHO) and Cardiac Magnetic Resonance (CMR).

Echocardiography is a simple, fast, cheap and safe imaging method^3^. In the last two decades, it has been the most frequently used technique to assess left ventricular (LV) structure and function in mice. New high-resolution instruments have now been designed, specifically to study small rodents, using high-frequency probes (30–50 MHz) with higher spatial resolution (30 µm), allowing more accurate assessment of the left ventricle^2,3^. However, the main limits of this method are its geometric assumptions, the acoustic window characteristics, probe positioning errors, and a highly subjective approach that makes a skilled sonographer indispensable^3^. Moreover, despite echocardiography is the routine imaging method used for non-invasive left ventricular function assessment in small rodents, a systematic critical evaluation of imaging quality and of the resulted data, is often lacking.

Cardiac Magnetic Resonance is the gold standard for non-invasive assessment of cardiac function in small rodents^4–6^. With the strength of its significant intrinsic contrast and not dependent on geometric assumptions, it allows the acquisition of contiguous slices of short-axis images along the entire length of the left ventricle, permitting accurate estimates of LV volumes (LVVs). Although CMR is the most reliable method, with the highest sensitivity and specificity to study the heart anatomy *in vivo*, it is expensive and fairly time-consuming compared to ECHO, which why it is not routinely used in large series of animals or for serially repeated exams^4–6^.

Several studies have proposed new ECHO approaches for assessing cardiac function, in order to avoid the limits of the standard left chamber quantification in small rodents^4,7–9^. One of these methods is the echocardiographic 3D reconstruction of the left ventricle^4^, which has been found to have good agreement with CMR for Left Ventricular Ejection Fraction (LVEF) calculation in several clinical studies^10^. However, it is not widely used, and is still not available in most of the experimental laboratories.

Another general crucial issue is that stringent and robust comparisons between ECHO and CMR in the setting of pharmacological preclinical research not specialized in imaging are still necessary.

Here we investigated whether an echocardiographic approach which allows acquiring serial short-axis images using a high-resolution ultrasound system allows a better agreement with CMR for LV function assessment in a mouse model of myocardial infarction.

## Methods

All supporting data are available within the article and upon reasonable request to the corresponding author.

### Animal use and care

Mice were housed at constant room temperature of 23 °C and relative humidity (60 ± 5%) with *ad libitum* access to food and water and fixed 12 hours light/dark cycle. Procedures involving animals and their care were conducted in conformity with the institutional guidelines at the Mario Negri Institute for Pharmacological Research IRCCS (Istituto di Ricovero e Cura a Carattere Scientifico) in compliance with national (D.lgs 26/2014; Authorization no. 19/2008-A issued March 6, 2008 by the Ministry of Health of Italy) and international (European Economic Community Council Directive 2010/63/UE; the National Institutes of Health *Guide for the Care and Use of Laboratory Animals*, 2011 edition) laws and policies. Procedures were reviewed and approved by the Mario Negri Institute Animal Care and Use Committee, which includes *ad hoc* members for ethical issues, and by the Italian Ministry of Health (Decreto no. 62/2012-B). Animal facilities meet international standards and are regularly checked by a certified veterinarian who is responsible for health monitoring, animal welfare supervision, experimental protocols, and review of procedures.

### Surgical procedures

A total of 25 C57BL/6J mice (body weight ranging 23–31 g) were anesthetized with isoflurane and permanent occlusion was induced by Coronary Artery Ligation (CAL), as previously described^11^. In 16 sham-operated mice (SHAM) surgery was done without CAL with the suture placed loosely around the left coronary artery. All animals received buprenorphine (0.1 mg/kg s.c. q12h) for one day as post-surgical analgesia and were followed for six weeks. Mortality was constantly recorded until six weeks after surgery. Mortality rate in CAL mice was 28% (n = 18 survived) while all SHAM mice (n = 16) reached the end of the follow up.

### ECHO: Image acquisition

Echocardiography was performed using a 30 MHz mechanical probe with a spatial resolution of 30 µm (VisualSonics, Vevo 770, Toronto, Canada) 6 weeks after CAL on mice anesthetized with isoflurane (0.5–1.5% in O_2_). Animals were positioned on a rail system for maintenance of the body temperature (37 °C ± 0.5 °C) and the probe position under electrocardiographic (EKG) and respiratory monitoring for the entire duration of the exam.

**Figure 1:**
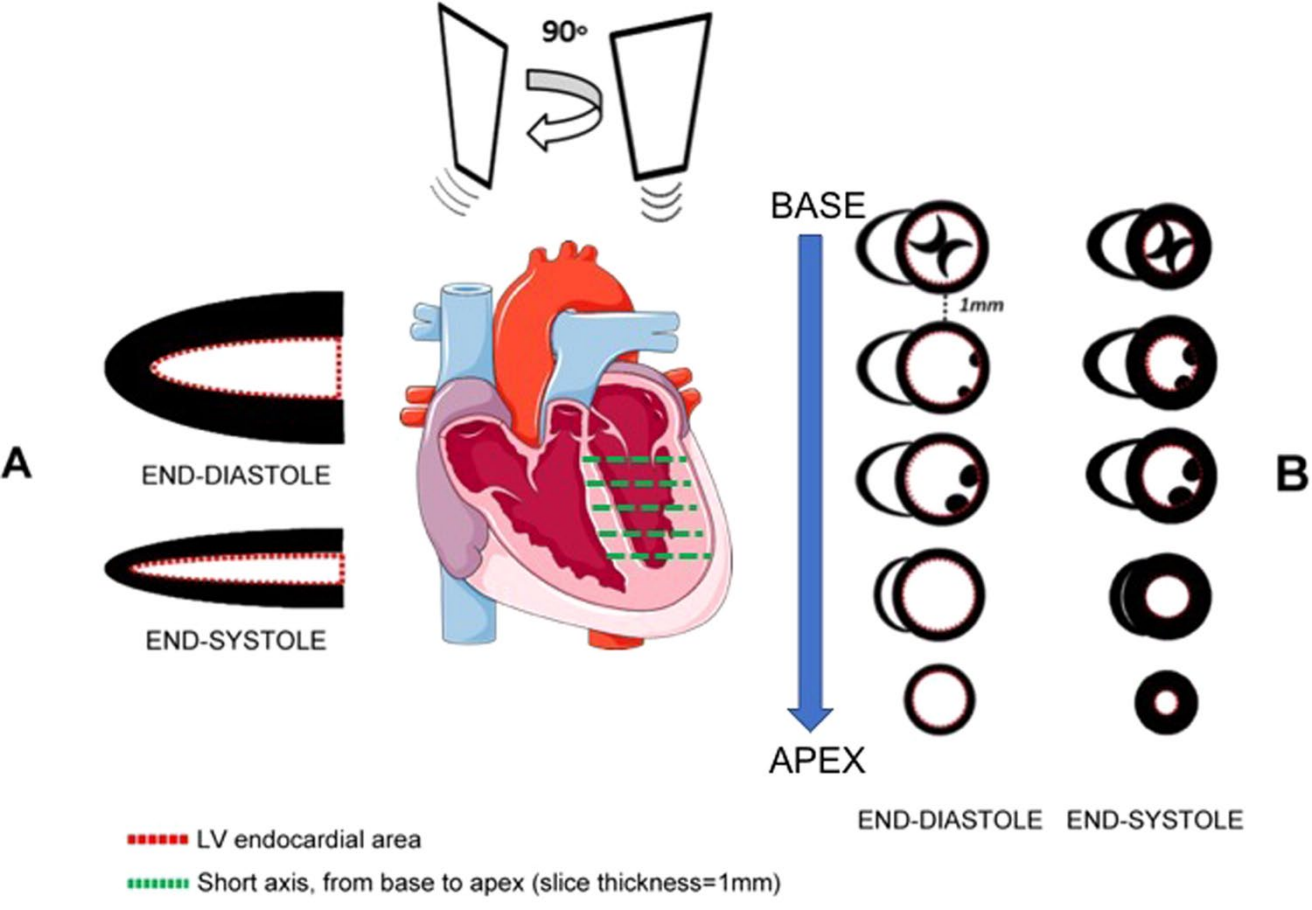
ECHO pLAX and ECHO pSAX methods. Schematic representation of ECHO pLAX (A) and ECHO pSAX (B) acquisition. (**A**) 2D ECHO cine-loops were taken from parasternal long axis view and endocardial diastolic and systolic areas were traced according to the maximal and minimal LV expansion. (**B**) transducer was rotated approximately 90 degrees clockwise from the parasternal long axis until left ventricle appeared circular. Serial cine-loops of 2D images from parasternal short axis view were acquired from base to apex by translating the ultrasound probe by 1 mm using a customized system. The figure was drawn using Servier Medical Art (https://smart.servier.com).

Parasternal long-axis (pLAX) B-mode image was acquired, optimizing the LV length for LV volume measurements and ejection fraction calculations (Fig. 1A). Parasternal short-axis (pSAX) 2D images of the left ventricle were recorded by translating the probe every 1 mm from the base to the apex (Fig. 1B). Cine loops containing five cardiac cycles (25–35 frames per cardiac cycle at a frame rate of 300–400/sec) were stored in Digital Imaging and COmmunications in Medicine (DICOM) format and measurements were taken off-line.

### ECHO: LV volumes and ejection fraction calculation

**Figure 2:**
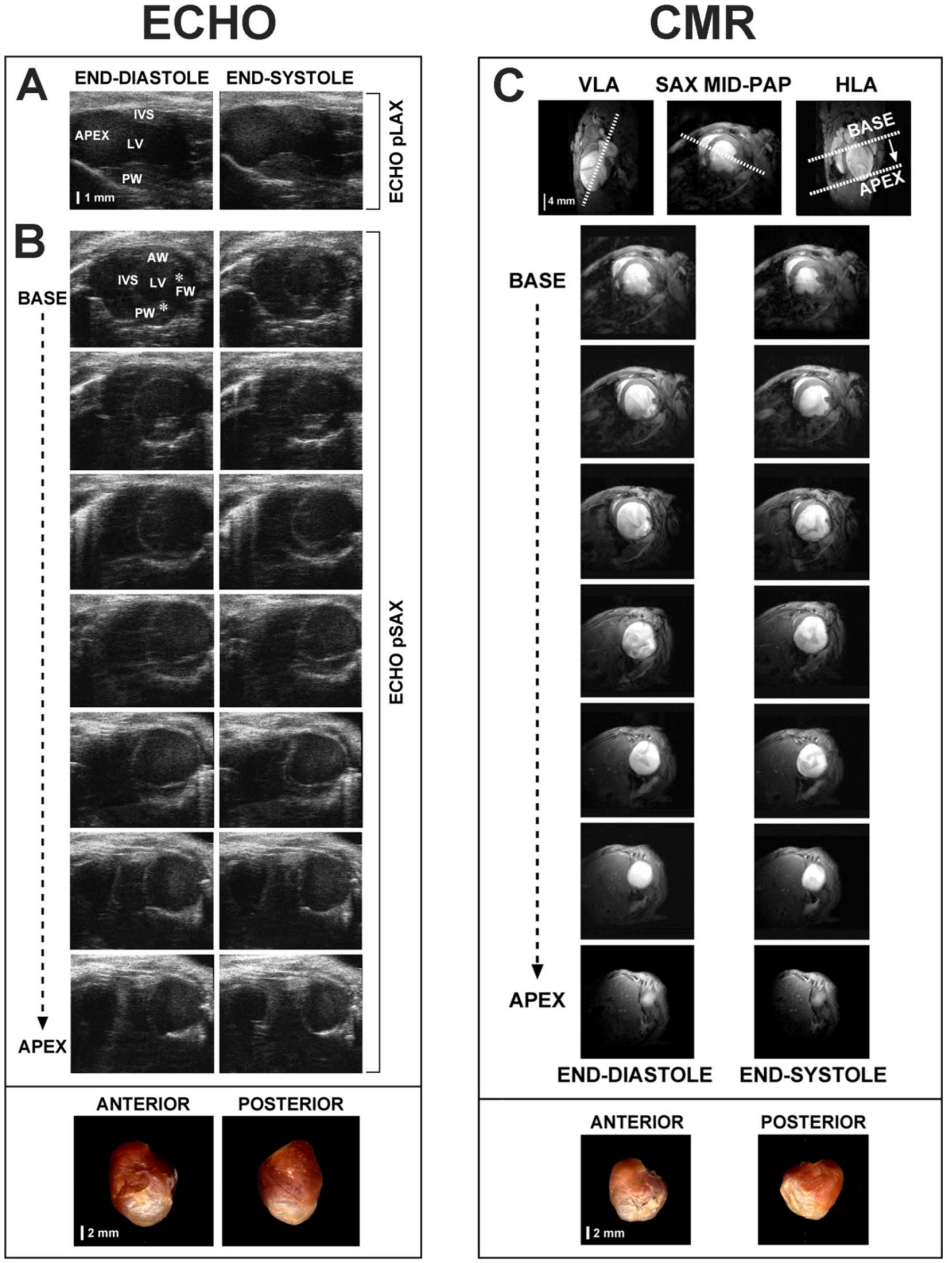
ECHO and CMR images. *In vivo* echocardiographic images of infarcted heart taken from 2D parasternal long axis (**A**) and from serial consecutive parasternal short axis (**B**) and used to apply the ECHO pLAX and the ECHO pSAX methods, respectively. Both echocardiographic methods were used to calculate LV volumes and ejection fraction and were compared to CMR (**C**). Corresponding *ex-vivo* photographs of anterior and posterior walls of the left ventricle in corresponding animals are presented in the bottom of the panel. AW, anterior wall; FW, free wall; IVS, interventricular septum; LV, left ventricle; PW, posterior wall; HLA, horizontal long axis; MID-PAP, mid-papillary level; VLA, vertical long axis. *Indicates papillary muscles.

Measurements were made off line by two sonographers (I.R., L.S.) blinded to experimental groups. LV endocardial areas were drawn manually in end-diastolic and end-systolic frames in images from pLAX and pSAX views. LV end-diastolic and end-systolic volumes (LVEDV and LVESV) were calculated using two methods: (1) a standard Single-plane modified Simpson (ComPACS software, Medimatic S.R.L, Genoa, Italy) from the pLAX view (ECHO pLAX, Fig. 2A); (2) from the pSAX view acquiring sequential short-axis 1 mm-thickness slices covering the entire left ventricle (ECHO pSAX, Fig. 2B). Diastolic and systolic endocardial areas were drawn manually in each slice and volumes were calculated with the formula used in CMR^12,13^ as follows: Σ(LVEDA) or Σ(LVESA)*slices thickness (LVEDA, end-diastolic area; LVESA, end-systolic area). Ejection fraction was calculated following the formula: (LVEDV-LVESV)/LVEDV*100. For each parameter, the mean of 3–5 consecutive measurements was calculated.

### CMR: Image acquisition

Cardiac Magnetic Resonance was done three days after ECHO on a horizontal bore 7 Tesla USR preclinical MRI system (BioSpec 70/30, BrukerBioSpin, Germany) with a shielded gradient insert (BGA 12, 400mT/m; rise time 110 ms). A linear volume coil and anatomically shaped surface coil were used to transmit/receive the magnetic resonance signals (linear volume coil inner diameter, 72 mm; surface coil inner diameter, 10 mm, Bruker BioSpin, Germany). After the localizer, scans (segmented double-gated FLASH imaging) were run to ensure correct positioning, tuning and matching the probe; slice-selective shimming and flip angle calibration were done manually before each experiment. Cine-MRI pulse-sequences were: matrix 256 × 128; FOV 40.0 × 20.0 mm; echo time, 2.98 ms; flip angle 15°; slice thickness, 1 mm; number of averages, 6; repetition time, 12 ms.

Mice, anesthetized by facemask with isoflurane (at the same concentration used in ECHO) and 0.3 L/min O_2_, were positioned in a purpose-built cradle and EKG electrodes were attached to the front paws. A pressure transducer for respiratory gating was positioned above the abdomen. A fiber optic probe was used to monitor the rectal temperature. EKG and respiratory signals were processed and displayed using a gating device and temperature was maintained at 37 ± 0.5 °C throughout the examination (SA Instruments, Stony Brook, NY Model 1025 MRI compatible physiological monitoring system).

After the image plane orientation from the coronal, sagittal and axial LV long-axis, a vertical long axis (VLA) was obtained and, orthogonal to it, adjusting orientation from a short axis slice at the mid-papillary muscle level, a horizontal long axis (HLA) was acquired followed by 1 mm serial short-axis slices covering the entire LV length (Fig. 2C). Six to eleven short-axis slices were acquired from base to apex. Sixteen frames per slice for one cine sequence were stored (two cardiac cycles) to ensure the acquisition of maximum and minimum LV expansion (end-diastole and end-systole). Images were exported in DICOM format and analyzed off-line.

### CMR: LV volumes and ejection fraction calculation

Two investigators (I.R., L.S.) blinded to the experimental conditions analyzed CMR recordings. End-diastolic and end-systolic parameters were measured in selected frames respectively according to the visual estimation of the maximal and minimal ventricular cavity. End-diastolic and end-systolic endocardial areas of each slice were traced manually. LV volumes and ejection fraction were calculated using the CMR formula.

All echocardiographic and CMR examinations were included in the analysis if they presented at least five distinct sequential short-axis slices where epicardial and endocardial borders were adequately visualized.

### *In vivo* measurement of infarct size

Infarct size was calculated *in vivo* by CMR considering the areas of akinesis. In the end-diastolic frames, the epicardial and endocardial circumferences were traced separately from the length of the infarcted tissue^5^. Three to five short-axis views were considered from the first slice that showed an akinetic segment to the last one to obtain a measurement of infarct size applying the formula: Σ (I_epi_/T_epi_ + I_endo_/T_endo_)*100]/n slices (T_epi_ and T_endo_ = total epicardial and endocardial circumference of left ventricle; I_epi_ and I_endo_ = epicardial and endocardial length of infarcted tissue). Infarct size is expressed as percentage of the left ventricle.

### Sample size calculation and statistical analysis

For sample size calculation, we assumed that the mean difference (µ), the standard deviation of difference (σ) and the predefined clinical limits of agreement (δ) between ECHO and CMR in LVEF values should not have been greater than −0.3 ± 8.2%, with 95% limits of agreement between −16.4 and 15.7; these values are based on our experience and are also considered clinically plausible^10^. According to the above statement, we calculated an approximate sample size of 19 mice per group in order to apply the Bland and Altman method with a non-central t-distribution assuming a two-side α error of 0.05% and a power of 80%^14^. In addition, considering a six weeks mortality rate of 30% in CAL mice, the number of animals to include in the CAL group was 25.

Data are expressed as mean ± standard error (SE). Differences between two groups were analyzed for statistical significance with an unpaired Student’s t-test. Correlations *(r)* between CMR and echocardiographic measurements and between infarct size and LV volumes and ejection fraction measured by ECHO and CMR were tested by Pearson’s method. Limits of agreement between imaging methods were established as the mean difference (bias) ±1.96 standard deviation (SD) of the differences, as described by Bland and Altman^15,16^. We calculated 95% limits of agreement for means and mean differences as 1.96*SD. Statistical significance was set at 5% (Graph Pad Prism 6.0).

The reproducibility of ECHO pLAX, ECHO pSAX and CMR for LV volumes and ejection fraction measurements was evaluated by calculating the intra-observer and inter-observer variability in both SHAM and CAL groups. Variability was defined as the absolute difference between the corresponding repeated measurements for each animal and expressed as percentage of their mean as represented in the following formula^17,18^: % variability = (measure 1-measure 2/average of measure 1 and measure 2)*100. The resulted data are expressed as mean ± SD.

## Results

### *In vivo* data

**Figure 3:**
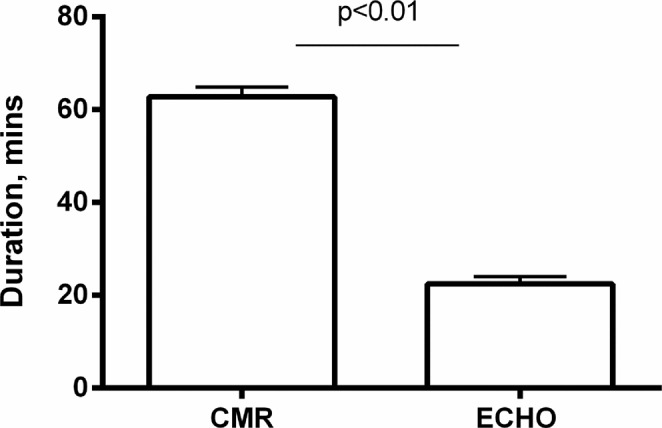
Acquisition time. Comparison between ECHO and CMR imaging duration considering all 34 animals. Data are expressed as mean ± SE. P value from Student’s t-test.

Six weeks after surgery body weight (BW) was not different in CAL and SHAM mice (Table 1). Although CMR total acquisition time was three-times longer than ECHO (CMR 68 mins *vs*. ECHO 23 mins, Fig. 3), the duration of the CMR and ECHO exams was similar in SHAM and CAL mice (CMR: 60 mins and 65 mins, ECHO: 23 mins and 22 mins, respectively). The heart rate did not differ either between the two groups (Table 2).

**Table 1:** *In vivo* data.

	SHAM	CAL
n	16	18
BW (g)	26.2 ± 0.8	26.6 ± 0.5
HW (mg)	109.4 ± 3.0	137.9 ± 4.4^#^
HW/BW	4.2 ± 0.1	5.2 ± 0.5^§^
Infarct size (%)		40.2 ± 3.3

Abbreviations: CAL, Coronary Artery Ligation; n, number of animals; BW, body weight; HW, heart weight. P value from Student t-test: ^#^p < 0.0001 vs. SHAM; ^§^p < 0.001 vs. SHAM.

**Table 2:** CMR and ECHO parameters in SHAM and CAL mice.

	SHAM	CAL	P values
**HR** (**bpm**)			
CMR	493 ± 12	463 ± 15	NS
ECHO	460 ± 19	444 ± 16	NS
**LVEDV** (**µl**)			
CMR	48.6 ± 6.0	108.1 ± 12.3	<0.01
ECHO pLAX	48.9 ± 1.7	102.3 ± 5.3	<0.01
ECHO pSAX	46.4 ± 2.4	112.0 ± 12	<0.01
**LVESV** (**µl**)			
CMR	16.0 ± 0.7	79.3 ± 11.6	<0.01
ECHO pLAX	12.9 ± 1.0	67.8 ± 4.8	<0.01
ECHO pSAX	16.4 ± 1.3	82.0 ± 11.4	<0.01
**LVEF** (**%**)			
CMR	67.1 ± 1.0	30.0 ± 2.8	<0.01
ECHO pLAX	73.9 ± 1.4	33.9 ± 2.1	<0.01
ECHO pSAX	63.6 ± 2.2	29.8 ± 2.3	<0.01

Data are shown as mean ± SE; Abbreviations: bpm, beats per minute. LVEDV, left ventricular end-diastolic volume; LVESV, left ventricular end-systolic volume; LVEF, left ventricular ejection fraction. P values from Student’s t-test.

In mice with CAL, infarct size ranged between 11.4% and 69.1% (mean value = 40.2 ± 3.3%, Table 1), LVEDV was 2.2 times greater in CAL compared with SHAM mice (p < 0.05), LVESV resulted 5.0 times greater and LVEF was 2.2-fold lower (p < 0.05). Mean values of LV parameters studied are presented in Table 2.

Heart weight and the ratio of heart weight to body weight were significantly higher in CAL than SHAM mice (+27%, p < 0.0001 and +24%, p < 0.001,) (Table 1).

### Agreement between ECHO and CMR

**Figure 4:**
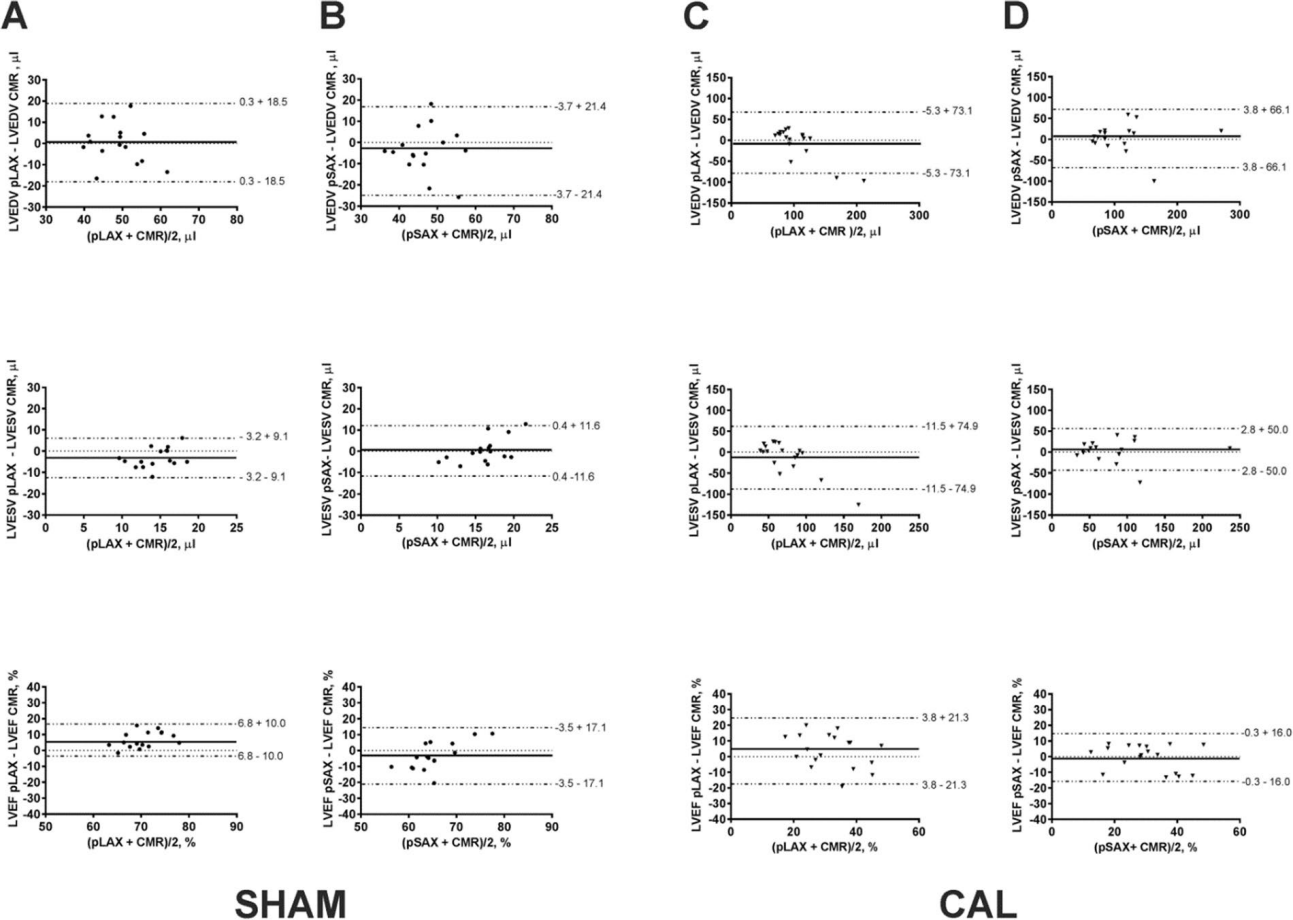
Bland and Altman plots for SHAM and CAL mice. The agreements between CMR and ECHO pLAX (**A**,**C**) and between CMR and ECHO pSAX (**B**,**D**) in SHAM and CAL mice are presented. See corresponding Table 3 for each mean difference. Single CMR and ECHO parameters are presented in Table 2.

In SHAM mice, the mean of differences between ECHO pLAX and CMR and ECHO pSAX and CMR for LVVs and LVEF was close to zero (Fig. 4A,B and Table 3). The smaller range of 95% limits of agreement when ECHO pLAX (−3.2–16.8%) was compared to CMR indicated that this method was more accurate than ECHO pSAX (−20.6–14.0%) for LVEF calculation in SHAM mice (Fig. 4A,B).

**Table 3:** Bland-Altman.

	SHAM		CAL	
	ECHO pLAX	ECHO pSAX	ECHO pLAX	ECHO pSAX
LVEDV (µl)	−0.3 ± 9.4	−3.7 ± 10.9	−5.3 ± 37.3	3.8 ± 33.7
LVESV (µl)	−3.2 ± 4.6	0.4 ± 5.9	−11.5 ± 38.2	2.8 ± 25.5
LVEF (%)	6.8 ± 5.1	−3.5 ± 8.7	3.8 ± 10.9	−0.3 ± 8.2

Agreement between ECHO methods and CMR. Data are shown as mean difference ± SD.

In CAL mice, ECHO pLAX underestimated LVVs (Fig. 4C), particularly LVESV (mean difference ± SD 11.5 ± 38.22 μL, Table 3). When ECHO pSAX was compared to CMR, the mean differences were close to zero for all studied parameters (Table 3) and the 95% limits of agreement showed a consistently smaller variation of the differences (Fig. 4D).

### Correlation between CMR and ECHO

As shown in Supplementary Fig. 1, in SHAM mice the correlation between LVVs and LVEF measured by ECHO and those obtained by CMR was not significant. In contrast, in CAL animals there was a significant linear correlation between LVVs and LVEF calculated by CMR with the values given by each of the ECHO methods used in the study.

### Inter- and intra-observer variability

The inter-observer and intra-observer variability in LVEDV, LVESV and LVEF calculation in SHAM and CAL mice for all studied imaging methods (Table 4) showed that all three methods are reproducible. In SHAM mice, a slightly higher percentage of inter-observer variability was observed for LVESV measurements in CMR and ECHO pLAX. In the CAL group, only small differences between observers were found and were higher for LVEDV measurements in CMR and ECHO pSAX and for LVESV in CMR.

**Table 4:** Variability.

	Inter-observer variability, %	Intra-observer variability, %
**EDV**		
ECHO LAX SHAM	2.3 ± 5.9	0.9 ± 2.0
ECHO SAX SHAM	−4.2 ± 1.4	0.2 ± 2.0
CMR SHAM	−0.2 ± 2.6	2.3 ± 1.5
**ESV**		
ECHO LAX SHAM	−4.4 ± 6.2	0.6 ± 3.9
ECHO SAX SHAM	−0.1 ± 8.9	−0.5 ± 2.2
CMR SHAM	−6.2 ± 8.0	1.9 ± 3.5
**LVEF**		
ECHO LAX SHAM	0.2 ± 6.6	0.7 ± 2.6
ECHO SAX SHAM	0.4 ± 3.6	0.8 ± 2.4
CMR SHAM	2.9 ± 3.7	−0.8 ± 3.3
**EDV**		
ECHO LAX CAL	0.3 ± 3.1	0.1 ± 4.4
ECHO SAX CAL	3.1 ± 3.7	1.9 ± 2.4
CMR CAL	3.7 ± 3.15	1.3 ± 3.2
**ESV**		
ECHO LAX CAL	1.2 ± 2.5	−0.4 ± 6.8
ECHO SAX CAL	1.8 ± 6.5	1.0 ± 3.9
CMR CAL	3.1 ± 5.4	3.1 ± 3.4
**LVEF**		
ECHO LAX CAL	2.9 ± 7.5	0.2 ± 4.7
ECHO SAX CAL	0.28 ± 8.2	2.6 ± 6.6
CMR CAL	2.0 ± 6.7	2.1 ± 5.5

Inter-observer and intra-observer variability in ECHO pLAX, ECHO pSAX and CMR for left ventricular parameters studied.

## Discussion

In the present study we have showed a close agreement between an accessible and fast echocardiographic method, the ECHO pSAX, and the gold standard CMR for LV volumes and ejection fraction calculation in mice with myocardial infarction. This finding has valuable implications in the experimental setting, particularly for longitudinal studies and animal welfare. In mice with a non-asymmetric left ventricle, ECHO pLAX measurements agree closely with those of CMR, thus this standard ECHO method might be confirmed as a fast and accurate approach for assessing systolic function in non-infarcted left ventricles.

This study showed, for the first time to our knowledge, the accuracy of a new high-resolution echocardiographic method of simple manual use for LV volumes and ejection fraction calculation in a large number of mice with myocardial infarction. Our data confirm previous results obtained by two independent studies with smaller number of mice and the use of an echocardiographic machine equipped with a 7/15 MHz linear-array probe^4,7^.

Echocardiography with high spatial resolution (30μm) along with an *ad hoc* instrument for LV scanning allowed contiguous cross-sectional segmentation of the left ventricle and more appropriate comparison between ECHO and CMR (Figs 1 and 2)

A customized hardware/software interface was developed in the last generation of echocardiographic machines in order to control the motor stage holding the ultrasound transducer and trigger the scanner to acquire and store data. This tool allowed for the automatic acquisition of finely sampled 2D short‐axis loops providing for a 3D assessment of the left ventricle.

As well known, the quantification of LV remodeling through LV volumes and ejection fraction measurements is useful for assessing the severity of LV injury, establishing the prognosis^7^ and studying the response to a specific treatment. Even if it is widely accepted that in an asymmetric left ventricle LV volumes and ejection fraction calculation is less accurate if derived from M-mode or from 2D long-axis view, these methods are the most frequently and routinely used in experimental settings.

Recently a 3D echocardiographic method has been published with a customized hardware/software interface developed to control a motor stage that holds the ultrasound transducer and trigger the scanner for the acquisition of serial sampled 2D short‐axis loops. This method was used to measure LV volumes and ejection fraction in mice with myocardial infarction but, to our knowledge, any correlation data and/or agreement was provided with CMR, the gold standard method to evaluate LV function^19^.

A recent study^20^ has compared ECHO and CMR in CAL mice by using echocardiographic serial slices obtained from parasternal long axis view. Interestingly the results showed an agreement in LV volumes and ejection fraction measurements between the studied methods which was similar to what has been found in our study, particularly for ejection fraction calculation, using the ECHO pLAX method (mean difference: 3.4% *vs*. 3.8%; 95% limits of agreement: −16.9–23.6 *vs*. −17.5–25.1, Fig. 4). However the agreement observed with ECHO pSAX in our study was closer (mean difference: −0.3%; 95% limits of agreement: −16–16, Fig. 4).

Our validation suggests that ECHO pLAX can be proposed as the elective method for LV volumes and ejection fraction calculation in symmetrical ventricles. LV ejection fraction in mice was slightly higher by ECHO pLAX compared to CMR (mean difference ± SD: 6.8 ± 5.1%, Table 3). This might be ascribed to long-axis peak velocities during systole which are slightly higher and rather homogeneous distributed in LV segments in mice compared to the radial and the rotational, according to Jung *et al*.^21^ by phase contrast CMR and/or to the difficulty in the identification of endocardial borders, particularly at apical sections. The echocardiographic underestimation of LV volumes compared to CMR is already known^22,23^. CMR is more accurate than ECHO in tracing end-diastolic areas with its better definition of the endocardium and easier exclusion of the papillary muscles and trabeculae. This might explain the wide limits of agreement between ECHO pSAX and CMR for LV volumes and ejection fraction calculation. We wonder whether these differences are acceptable: similar results were reported in humans where the limits of agreement for ejection fraction were −18.1–8.3% between ECHO (measured from apical 4-chamber and 2-chamber, views applying the biplane modified Simpson’s method) and CMR^23^ and even greater in rats when measurements were taken from the 2D parasternal long-axis and from a single 2D short-axis at the mid-papillary level^9^.

Bland and Altman analysis confirmed that the accuracy of ECHO pSAX, compared to ECHO pLAX, was closer to CMR. Moreover, since ECHO is 3-fold faster (Fig. 3) and cheaper (Supplementary Table 1) than CMR, even using a self-gating acquisition scheme^24^, ECHO pSAX should be a reliable alternative for studying the LV systolic function in mice with myocardial infarction. It is clear that ECHO pSAX can be only used in anesthetized mice and echocardiographic machine must be equipped with high-resolution ultrasound and a mechanical system to move the probe in order to obtain sequential slices. As mentioned before, similar approach was recently used by O’Connor DM and colleagues^19^ who measured LV volumes and ejection fraction in mice with reperfused myocardial infarction using an ultrasound system, with the same resolution as we used but equipped with a customized hardware/software interface for automatic acquisition.

It is debated whether ECHO pLAX is a valuable method for assessing LV function in experimental models of myocardial infarction^4^. We found a small mean difference and acceptable 95% limits of agreement between ECHO pLAX and CMR for LV ejection fraction but not for LV volumes (Fig. 4). These results suggest that without a high-resolution ultrasound platform, the ECHO pLAX method can be used for LV ejection fraction calculation in mice with myocardial infarction, but LV volumes will be underestimated.

We found no statistical significance in the correlation between ECHO pLAX and CMR measurements in SHAM mice possible because the values were concentrated in a very narrow range (Supplementary Fig. 1).

We wondered if ECHO pSAX method could be applied to other species used as models in cardiovascular research. Two and four apical chambers views are recommended by the echocardiographic guidelines to accurately assess LV volumes and ejection fraction (particularly in asymmetric ventricles) by using the biplane method of disk summation technique^25^. Here we proposed the ECHO pSAX method because of the impossibility to obtain standardized and non-foreshortened apical views in mice, the experimental myocardial infarction most used animal model. Based on our experience, ECHO pSAX method could be used to evaluate rats, whereas its applicability on other species such as rabbits and feline would need further analysis. In large models like dogs and pigs, the use of two and four apical chambers views has been reported by our group and others^26,27^, and is fully accepted as standard method for LV volumes and ejection fraction calculation. However, our method could be an alternative to use in those animals with a non-optimal apical window.

As already mentioned, scan time during CMR may explain the higher LV volumes observed. In order to expedite CMR procedure, in the last few years, new coils with higher signal-to-noise ratios (SNR) using parallel elements and/or cryocooling techniques have been developed^28^. Only one study in rats^29^ has used a 4-element array. Clearly due to the different cardiac dimensions between rats and mice, the SNR and the image quality obtained in that study cannot be compared with that obtained in our experimental setup. In the present study, the CMR acquisition could have been shortened by using an under sampling of the k-space; however, this procedure reduces the SNR of the image and could have produced image distortions. The prospectively triggered acquisition on EKG signal is also time-consuming. The introduction of cardiac and respiratory self-gated (Intragate®) acquisition with retrospective reconstruction of the cardiac cycle^24^ could have shortened the CMR acquisition. Unfortunately, it may be optimal for assessing normal cardiac hearts but may give an inaccurate analysis in infarcted animals. First, the decrease in SNR and in the contrast-to-noise ratio between blood and myocardial wall may be challenging in the identification of the endocardial borders of the thin infarcted wall; second it may not be able to consider the possible arrhythmias and EKG disorders that can arise in CAL mice. Another issue to underline is that we saved 16 frames per cardiac cycle for accurate detection of end-diastole and end-systole phases, and this latter takes part in the total acquisition time.

## Study Limitations

In mice with severe impairment of LV function, a reduced inflow effect may lower the contrast-to-noise ratio affecting the accuracy of our measurements, as described by other authors^30^. However, a significant correlation was found between myocardial infarction size measured by CMR and LV volumes and ejection fraction measured by all methods studied with a positive association with LV volumes and a negative association with ejection fraction (Supplementary Fig. 2).

Since mice with large myocardial infarction are likely to be affected by repeated anesthesia^2^, we adopted a three days interval after ECHO before CMR to allow for the reestablishment of vital parameters and ambulatory behavior and this could have affected the actual agreement between the two imaging methods^31^.

We are not able to suppose whether ECHO pSAX method may improve the echocardiographic assessment of LV remodeling in different experimental models without marked variations in LV geometry (i.g hypertrophic cardiomyopathies -such as aortic banding or transgenic mice-, HFpEF models, etc). In non-dilated left ventricles, the identification of the apical end-systolic endocardial border was difficult due to the superimposition of local irregularities of the mural endocardium. We suppose that this issue is the reason why ECHO pLAX method showed better agreement with CMR.

LV mass is also an overall important variable of LV remodeling. However, several works^32,33^, have showed significant correlation between echocardiographic LV mass and crude LV weight in normal and post-aortic banding mice but not in myocardial infarction models. In dilated infarcted hearts, in fact, hypertrophy may be severe but may be masked by the thinning of the left ventricular wall thus the actual low LV weight may mask the presence of hypertrophic segments explaining the absence of correlation between LV mass and LV weight in myocardial infarction models. For this latter reason we did not include LV mass as variable in our study.

In the last few years hardware has made great improvements in CMR field. The new cryocoils, with their gain in the SNR, between 3.0–5.0^34^, have greatly reduced the CMR scanning time (namely 6 times faster) but this is still a costly hardware upgrade which is not always feasible.

## Conclusions

LV volumes and ejection fraction measured by ECHO pSAX and CMR were very similar. This result has important practical implications in experimental settings, since ECHO is more readily available. However, ECHO pLAX remains a reliable method for LV function assessment of symmetrical left ventricle and for LV ejection fraction calculation in infarcted mice.

## Supplementary information

ONLINE SUPPLEMENTARY MATERIAL
