# Supplementary material for: A novel echocardiographic method closely agrees with cardiac magnetic resonance in the assessment of left ventricular function in infarcted mice
Source: Sci Rep. 2019 Mar 5;9:3580. doi: 10.1038/s41598-019-40393-0 (PMC6400943; doi:10.1038/s41598-019-40393-0)
Supplement: Supplementary file 1 — ONLINE SUPPLEMENTARY MATERIAL [file 41598_2019_40393_MOESM1_ESM.docx]

**SUPPLEMENTARY MATERIAL**

**A novel echocardiographic method closely agrees with cardiac magnetic resonance in the assessment of left ventricular function in infarcted mice**

Ilaria Russo^1*^, Edoardo Micotti^2^, Francesca Fumagalli^1^, Michela Magnoli^1^, Giuseppe Ristagno^1^, Roberto Latini^1^, Lidia Staszewsky^1^

**SUPPLEMENTARY TABLE AND TABLE LEGEND**

|  | **CMR** | **ECHO pLAX** | **ECHO pSAX** |
| --- | --- | --- | --- |
| **Total costs (USD)** | 399 | 137 | 171 |
| **Relative cost difference vs. CMR (%)** | - | -66 | -57 |
| **Total saving vs. CMR (USD)** | - | 262 | 228 |

**Supplementary Table-1: Costs, relative cost difference and savings between imaging procedures.** Based on our intramural costs, CMR is the most expensive imaging service accounting 399 USD whereas echocardiography including a complete structural and functional analysis of left ventricle, using pLAX for LVVs and ejection fraction calculation, costs 137 USD. ECHO pSAX results only 34 USD more expensive than ECHO pLAX (171 USD). Costs are calculated considering the time for animal preparation, induction of anesthesia, images acquisition and post-procedure animal care. ECHO pLAX acquisition is the most convenient analysis (-66% vs. CMR) although not much convenient compared to ECHO pSAX considering its lower accuracy.

**SUPPLEMENTARY FIGURES AND FIGURES LEGENDS**

**
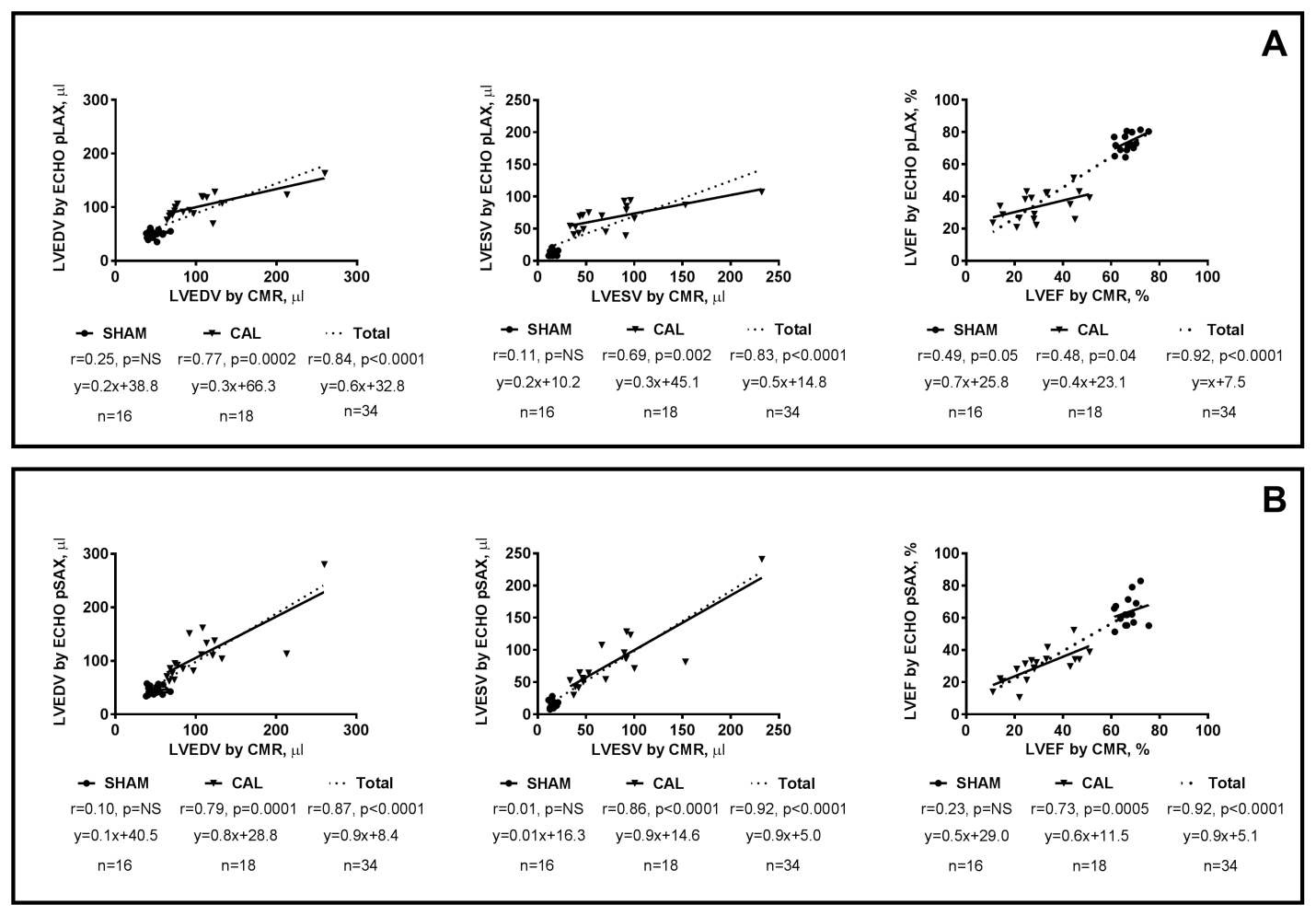
**

**Supplementary Figure-1:** **Correlation between CMR and ECHO measurements.** Plots of LV volumes and ejection fraction in SHAM and CAL mice are presented. CMR compared to ECHO pLAX (A) or to ECHO pSAX (B). r indicates Pearson correlation coefficient; NS, not significant; n indicates the number of animals per group. Other abbreviations are defined in the text.

**
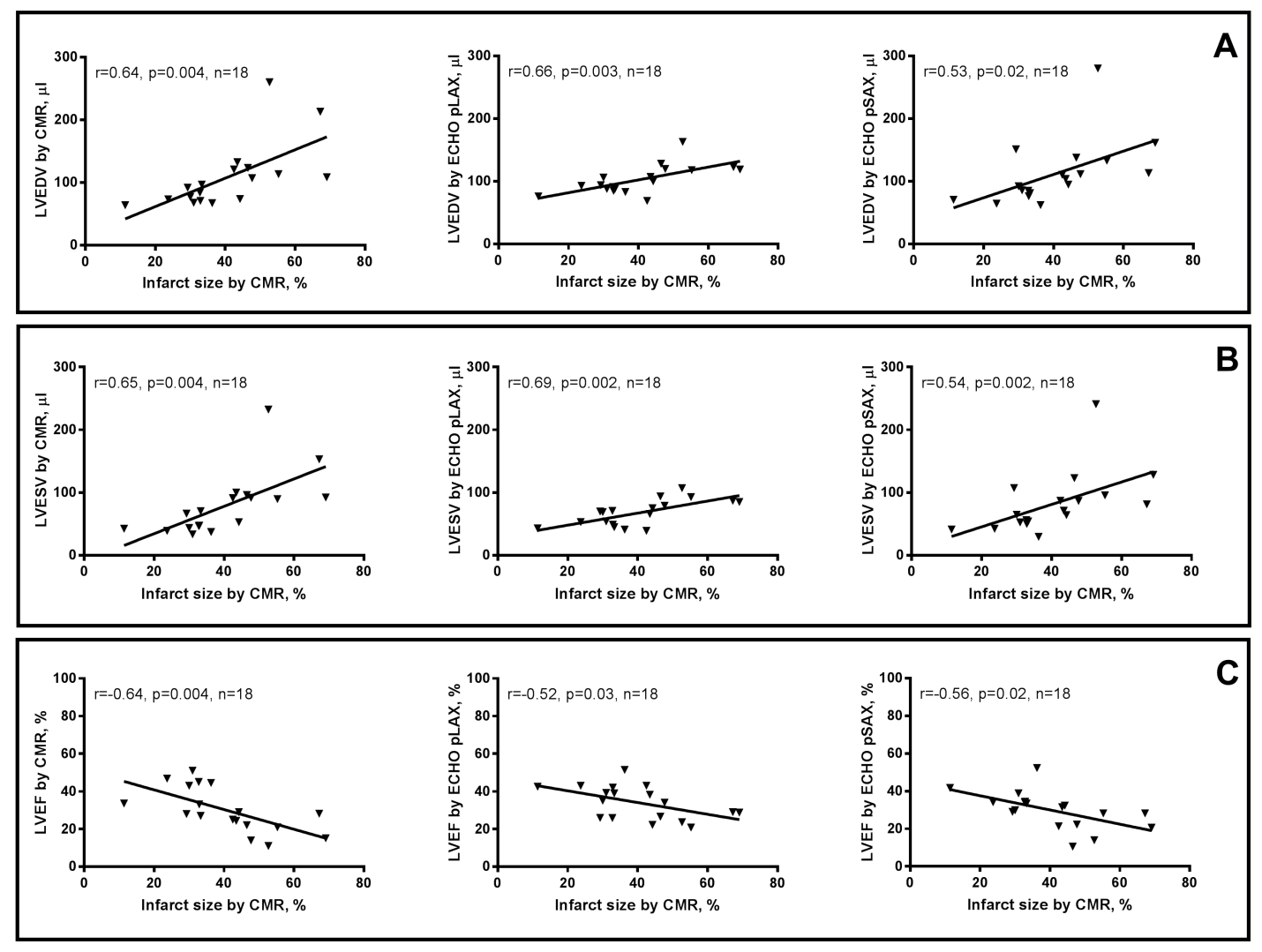
**

**Supplementary Figure-2: Correlation between LVEDV (A), LVESV (B), LVEF (C) and infarct size.** r indicates the Pearson correlation coefficient; n indicates the number of animals per group. Other abbreviations are defined in the text.
